# A partial articular‐sided supraspinatus tear caused by the biceps tendon: A novel etiology of internal impingement

**DOI:** 10.1002/ccr3.4044

**Published:** 2021-06-24

**Authors:** Alexander Rosinski, James L. Chen, Patrick J. McGahan

**Affiliations:** ^1^ San Francisco Orthopaedic Residency Program San Francisco CA USA; ^2^ Advanced Orthopaedics and Sports Medicine San Francisco CA USA

**Keywords:** internal impingement, long head of the biceps, rotator cuff tear

## Abstract

Impingement of the LHB can directly lead to articular‐sided supraspinatus tears. When pain persists despite arthroscopic debridement, we recommend taking the arm out of traction intraoperatively and placing it in the 90‐90 position.

## Graphical abstract

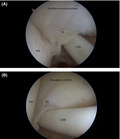

## 1 INTRODUCTION

Our patient is a 19‐year‐old female who presented with left shoulder pain. Diagnostic arthroscopy revealed an articular‐sided supraspinatus tear. When the shoulder was taken out of traction and placed in 90 degrees of abduction and external rotation, we visualized the biceps tendon directly impinging the undersurface of the supraspinatus tendon.

As shoulder arthroscopy progressed, surgeons noticed an association between partial articular‐sided rotator cuff tears and superior labral lesions.^1^, ^2^ It has been proposed that this pathology is caused by contact between the posterior‐superior margin of the glenoid and the humeral head when the arm is abducted and externally rotated.^1^, ^3^ This phenomenon has been labeled “Internal Impingement” as it is believed to result in impingement of the posterior‐superior labrum and the articular side of the supraspinatus tendon.^1^ Internal impingement is classically associated with throwing and other repetitive overhead athletic activities, particularly in the late cocking and early acceleration phases.^4^ Over time, repetitive loading at the limits of the functional arc of motion leads to predictable osseous and soft‐tissue adaptations which have been associated with internal impingement. Several pathological conditions may result including articular‐sided rotator cuff tears, labral tears, biceps tendonitis, and anterior shoulder instability.^5^

Over time, additional theories have emerged to explain the common association between articular‐sided rotator cuff tears and superior labral lesions. Burkhart et al proposed that these lesions develop over time due to the excessive shear and torsional forces placed on the shoulder by repetitive abduction and external rotation.^4^ Andrews et al proposed that repetitive eccentric loading leads to similar pathology.^6^ Although the true etiology of these lesions remains the subject of debate, the concept of internal impingement remains an important framework for understanding the painful shoulder in an overhead athlete.

The prevalence of internal impingement has not been well established due to the constellation of findings that characterize this condition and its strong association with other pathological lesions of the shoulder.^5^ However, several authors have identified internal impingement as a leading cause of partial articular‐sided rotator cuff tears in throwing athletes.^5^, ^7^ The purpose of this case report is to describe a novel etiology of internal impingement between the biceps tendon and the undersurface of the supraspinatus tendon leading to a symptomatic articular‐sided rotator cuff tear. The patient's symptoms persisted despite initial treatment with arthroscopic debridement and subacromial decompression. However, subsequent treatment with arthroscopic debridement and open subpectoral biceps tenodesis was successful and the patient has remained asymptomatic at latest follow‐up. To the authors' knowledge, no previous study has identified the biceps tendon as a cause of internal impingement.

## 2 CASE HISTORY

A 19‐year‐old right‐handed female presented to our clinic with a chief complaint of left shoulder activity‐related pain of approximately 6 months' duration. She subsequently underwent 6 months of physical therapy and received two subacromial cortisone injections with minimal relief. On physical examination, she reported pain over the anterior and lateral shoulder. She had pain with resisted supraspinatus testing but full strength. She also had moderate discomfort with abduction and external rotation (posterior impingement sign). There was mild tenderness to palpation over the biceps tendon in the groove as well. She had full range of motion and no anterior or posterior shoulder instability. Subscapularis and infraspinatus tests were normal and she had a negative O'Brien's test, negative jerk test, and negative subacromial impingement test. There was no scapular dyskinesis or glenohumeral internal rotation deficit (GIRD). The remainder of her exam was unremarkable.

An MRI showed a partial supraspinatus tendon tear and moderate tendinosis of the intra‐articular portion of the long head of the biceps (LHB). There was no evidence of anterior or superior labral tear. The upper border of the subscapularis tendon was intact. She opted for surgical management after a thorough discussion of operative and nonoperative treatment options.

### 2.1 Surgical treatment

**FIGURE 1:**
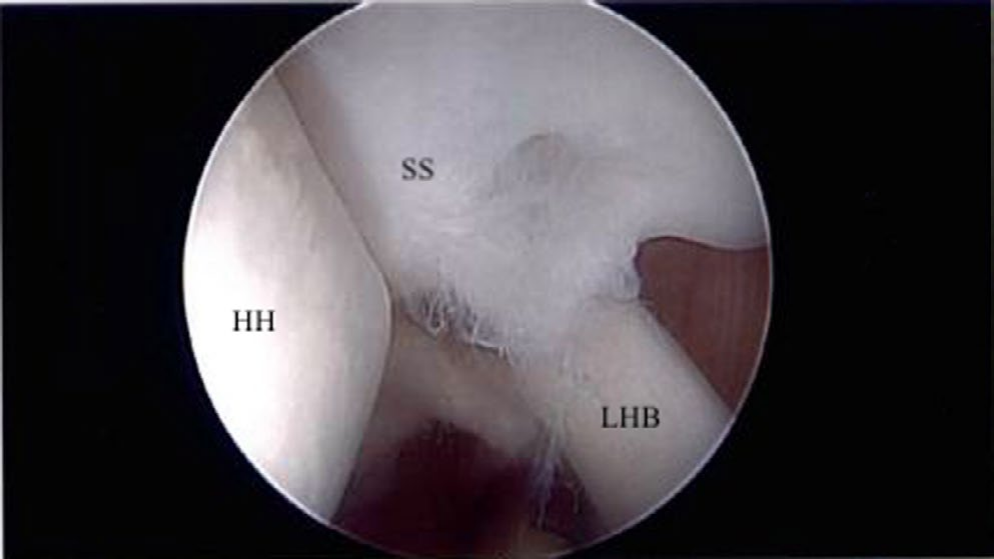
The patient's first diagnostic arthroscopy of the left shoulder revealed a 1 cm (medial‐lateral) by 5 mm (anterior‐posterior) lesion of the undersurface of the anterior aspect of the supraspinatus tendon. SS, supraspinatus tendon; LHB, long head of the biceps; HH, head of the humerus

Left shoulder arthroscopy was performed in the lateral decubitus position with 10 pounds of longitudinal traction. Diagnostic arthroscopy revealed a 1 cm (medial‐lateral) by 5 mm (anterior‐posterior) lesion of the undersurface of the anterior aspect of the supraspinatus tendon (Figure 1). There was mild posterior‐superior labral fraying, but no significant labral pathology. Mild biceps tendinitis was observed as the tendon was pulled into the joint, but its anchor on the superior labrum was intact. There was mild subacromial bursitis. There was also no evidence of an anterior labral tear or subscapularis tear. We performed an arthroscopic debridement of the rotator cuff and a subacromial decompression. Biceps tenodesis was not performed as there was no evidence of a superior labral tear, significant bicipital tendonitis, medial subluxation of the tendon, or concurrent subscapularis tear.^8^, ^9^

Her postoperative course was uneventful and she quickly regained full range of motion and strength. However, she continued to have similar activity‐related shoulder pain at 6 months of follow‐up. Physical examination demonstrated identical findings to her preoperative exam 6 months earlier. She was unable to return to collegiate swimming and water polo. After a thorough discussion, she returned to the operating room for a left shoulder diagnostic arthroscopy with possible rotator cuff repair, labral repair, and open biceps tenodesis.

**FIGURE 2:**
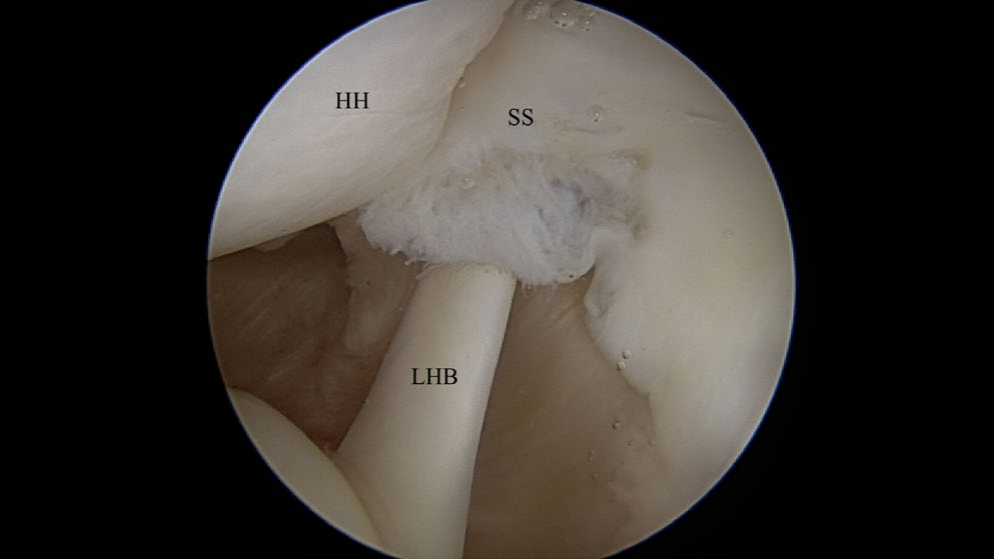
The patient's second diagnostic arthroscopy similarly revealed a 1 cm (medial‐lateral) by 5 mm (anterior‐posterior) lesion of the undersurface of the anterior aspect of the supraspinatus tendon. SS, supraspinatus tendon; LHB, long head of the biceps; HH, head of the humerus

**FIGURE 3:**
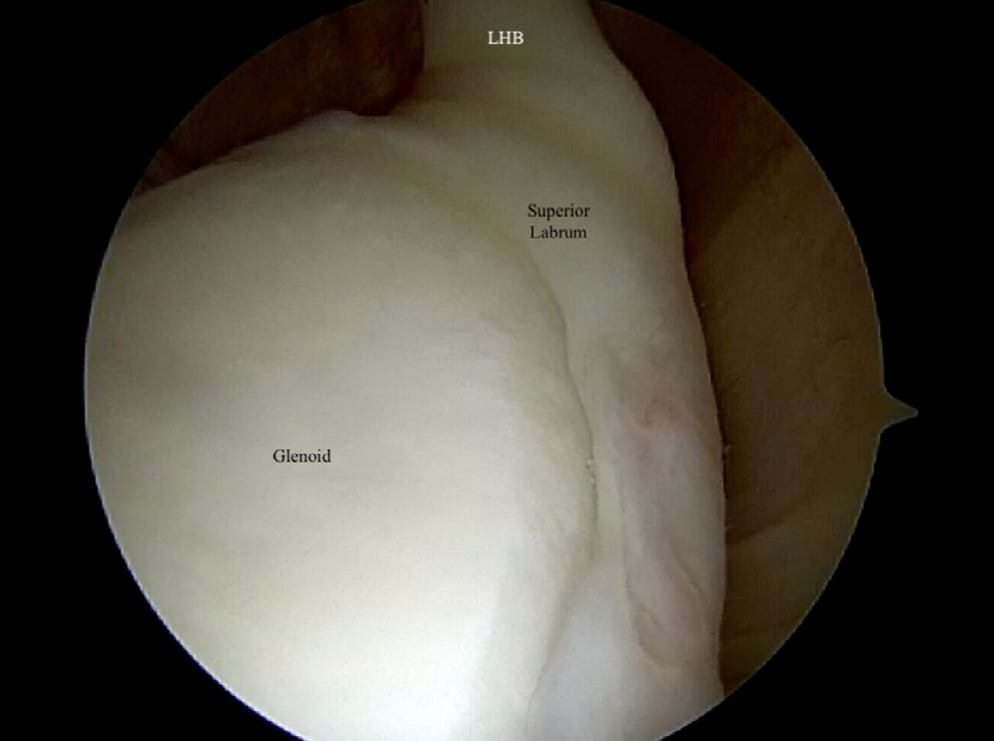
Diagnostic arthroscopy demonstrated mild posterior‐superior labral fraying, but no significant labral pathology. There was evidence of mild biceps tendinitis but its anchor on the superior labrum was intact. LHB, long head of the biceps

**FIGURE 4:**
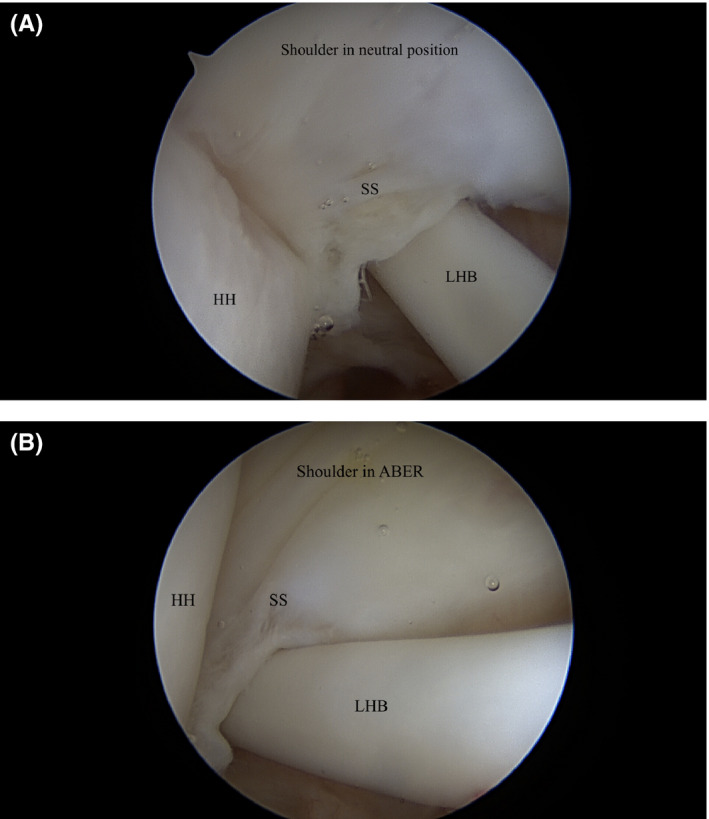
A, Diagnostic arthroscopy of the shoulder in neutral position. B, The biceps tendon directly impinges the undersurface of the supraspinatus tendon when the shoulder is abducted and externally rotated (ABER). The LHB also assumes a more vertical and posterior angle in this position. SS, supraspinatus tendon; LHB, long head of the biceps; HH, head of the humerus

Diagnostic arthroscopy once again arthroscopy revealed a 1 cm (medial‐lateral) by 5 mm (anterior‐posterior) lesion of the undersurface of the anterior aspect of the supraspinatus tendon (Figures 2 and 3). There was mild posterior‐superior labral fraying, but no significant labral pathology. There was mild biceps tendinitis as the tendon was pulled into the joint, but its anchor on the superior labrum was intact. There was no evidence of an anterior labral tear or subscapularis tear. At this point, the shoulder was taken out of traction and dynamic testing under direct arthroscopic visualization was performed. The arm was placed in a position of 90 degrees of abduction and 90 degrees of external rotation. To our surprise, we did not discover impingement between the supraspinatus tendon and the posterior‐superior glenoid. The articular side of the rotator cuff would not contact the posterior‐superior labrum. Instead, we visualized the biceps tendon directly impinging and abrading the undersurface of the supraspinatus tendon (Figure 4B). The biceps tendon fell directly into the articular‐sided tear of the supraspinatus tendon during abduction and external rotation. As a result, we elected to proceed with subpectoral biceps tenodesis to prevent further impingement.

The patient wore a shoulder sling for 4 weeks postoperatively and was instructed to avoid active biceps flexion. She began light resistive biceps strengthening at 8 weeks postoperatively and progressed to full activity at 3 months postoperatively. At 6 months, she had regained full strength and range of motion and had returned to competitive swimming and water polo. She has remained asymptomatic at 2 years of follow‐up.

## 3 DISCUSSION

Previous biomechanical and clinical studies have demonstrated the concept of internal impingement.^1^, ^2^, ^3^, ^4^ In abduction and external rotation, the humeral head often contacts the superior glenoid, resulting in impingement of the articular side of the supraspinatus tendon and the superior labrum.^1^ This impingement, in turn, can potentially lead to pathologic lesions of the rotator cuff and labrum.^1^, ^2^ Although alternative explanations for this pathology exist,^4^, ^6^ this theory has served as a useful conceptual framework for understanding pathology commonly found in the overhead athlete. The purpose of this case report is to describe an alternative etiology of internal impingement involving impingement of the biceps tendon on the undersurface of the supraspinatus tendon.

The association between partial articular‐sided rotator cuff tears and superior labral lesions is well documented in the literature.^1^, ^2^, ^6^, ^10^ It should be noted, however, that not all partial articular‐sided rotator cuff tears are accompanied by superior labral lesions.^11^ The fact that not all articular sided rotator cuff tears are accompanied by labral lesions leads us to believe that there are alternative causes of articular‐sided rotator cuff tears other than internal impingement. Over time, additional theories have emerged to explain the common association between articular sided rotator cuff tears and superior labrum lesions. Burkhart et al proposed that these lesions can develop over time due to the excessive shear and torsional forces cause by repetitive abduction and external rotation.^4^ Andrews et al proposed that repetitive eccentric loading can similarly lead to this pathology.^6^ As demonstrated in this case report, we believe that direct impingement of the biceps tendon on the undersurface of the rotator cuff can lead to articular‐sided rotator cuff tears.

Posterior biceps instability and friction may represent an under‐diagnosed and under‐recognized source of internal impingement. This may especially be true among patients whose symptoms persist despite adequate treatment of concomitant pathology. Partial articular‐sided rotator cuff tears and type‐1 labral lesions are typically treated nonoperatively with good results.^12^ When conservative treatment fails, arthroscopic debridement has been the standard of care.^13^ However, some studies have reported suboptimal results with this treatment approach.^10^, ^14^ When partial tears of less than 50% thickness are treated with arthroscopic debridement, the incidence of progression to full‐thickness tears ranges from 6.5% to 34.6% in the literature.^13^ Our experience reflects these findings as we have observed high rates of persistent symptoms in our practice with debridement alone. This observation has led us to search for other potential pain generators in the shoulder that may be overlooked. The biceps tendon as a source of pain and pathology in the shoulder has been well described.^15^ There are three primary sources of biceps‐related shoulder pain: bicipital tendonitis, traction on a Type‐2 SLAP tear, and biceps instability. Each of these conditions can be treated effectively with biceps tenodesis. In the case presented in this report, there was no evidence of significant bicipital tendonitis or labral detachment.

Multiple anatomic changes and pathologic conditions contribute to biceps instability in throwing athletes. Increased external rotation with repetitive throwing is achieved by shoulder adaptations including anterior capsule laxity and posterior capsule tightening.^16^, ^17^ Laxity of the superior glenohumeral and coracohumeral ligaments, which form the “biceps pulley” along with the supraspinatus and subscapularis, may contribute to biceps instability. Braun et al demonstrated that tears of both the anteromedial and posterolateral pulley are associated with biceps subluxation and dislocation from the bicipital groove.^18^ Acute trauma can also cause biceps pulley lesions.^19^ Swimmers such as our patient often have an element of underlying shoulder laxity.^20^, ^21^ Our patient's history of competitive swimming and water polo may have contributed to posterior biceps instability and the resulting internal impingement seen intraoperatively (Figure 4B).

In addition, abduction and external rotation in the late cocking phase of throwing is known to shift the biceps tendon posteriorly.^4^ In this position, the biceps tendon may assume a more vertical and posterior angle (as was observed in our patient during diagnostic arthroscopy in the 90‐90 position). Burkhart et al attributed the “peel‐back phenomenon” seen intraoperatively in patients with SLAP lesions to this posterior shift in biceps vector forces.^22^ Of note, the patient presented in this case report had some evidence of labral fraying, but the biceps anchor was intact. Although the “peel‐back phenomenon” was not observed, tension on the intra‐articular biceps tendon in the late cocking phase may have resulted in posterior displacement and impingement on the rotator cuff without fully disrupting its anchor.

Several clinical studies have demonstrated an association between rotator cuff tears and biceps instability.^8^, ^23^, ^24^, ^25^, ^26^ The LHB is stabilized by a sling formed by the supraspinatus and subscapularis tendons proximal to the bicipital groove.^27^ Therefore, rotator cuff tears may increase and alter mechanical loading on the LHB and lead to progressive deterioration.^23^, ^24^ LHB lesions have been reported in 29%‐86% of patients with rotator cuff tears.^8^, ^23^, ^24^, ^25^, ^26^ In a prospective study of 200 arthroscopic rotator cuff repairs, LHB subluxation or dislocation was observed in 45% of patients.^23^ Posterior LHB instability was commonly observed with anterior supraspinatus tendon tears.^16^

The are several weaknesses of this case report. First, this is an isolated case and it remains to be seen whether this approach is valid when applied to a larger patient population. Second, the patient underwent an additional debridement at the time of her second surgery, which may have contributed to the positive results. Third, biceps tenodesis is an effective treatment for biceps pathology. It is possible that the rotator cuff lesion was incidental and that the true pathology was intrinsic to the biceps tendon.

In summary, we present an interesting case report of a small, partial articular‐sided supraspinatus tendon tear initially treated with arthroscopic debridement (Figure 1). Unfortunately, the patient did not improve with simple debridement. In addition, the tear was relatively small and was not felt to be large enough to be causing significant pain on its own. This led us to search for an alternative explanation for the patient's shoulder pain. During arthroscopy, we observed direct impingement of the biceps tendon on the undersurface of the rotator cuff as the arm was abducted and externally rotated (Figure 4B). In addition, the remaining rotator cuff beyond this site of impingement was intact. This led us to perform an open biceps tenodesis. The patient recovered fully from this operation and is pain free at latest follow‐up.

## 4 CONCLUSION

In this case report, we propose a novel biomechanical source of internal impingement: direct impingement of the biceps tendon on the undersurface of the supraspinatus tendon. We believe that impingement of the LHB can either directly lead to, or potentially aggravate, articular‐sided tears of the supraspinatus tendon. This proposed mechanism of impingement may help explain why debridement alone can produce suboptimal results.^10^, ^13^, ^14^ In cases where pain persists despite arthroscopic debridement, we recommend performing a biceps tenodesis. In addition, we recommend taking the arm out of traction during arthroscopy and placing it in the 90‐90 position. If the biceps tendon can be seen directly abrading the supraspinatus tendon, we recommend primary biceps tenodesis.

## CONFLICT OF INTEREST

Dr Chen is an educator for Arthrex Inc The authors declare no conflicts of interest related to the subject of this article. No outside funding or grants were received for this study. Alexander Rosinski, MS: This author, their immediate family, and any research foundation with which they are affiliated did not receive any financial payments or other benefits from any commercial entity related to the subject of this article. James Chen, MD, MPH: This author, their immediate family, and any research foundation with which they are affiliated did not receive any financial payments or other benefits from any commercial entity related to the subject of this article. Patrick McGahan, MD: This author, their immediate family, and any research foundation with which they are affiliated did not receive any financial payments or other benefits from any commercial entity related to the subject of this article.

## ETHICAL APPROVAL

Informed consent was obtained from the patient regarding the report and publication of the case in an anonymous way.
